# Advanced Age in Sinus Surgery: Diminished Symptom Gains but Enhanced Surgical Durability in Chronic Rhinosinusitis

**DOI:** 10.1002/ohn.70043

**Published:** 2025-10-14

**Authors:** Ahmad A. Mirza, Hussam A. Senan, Juan C. Hernaiz‐Leonardo, Osama A. Marglani, John M. Lee, Ahmad R. Sedaghat, Amin R. Javer

**Affiliations:** ^1^ Department of Otolaryngology–Head and Neck Surgery, Faculty of Medicine in Rabigh King Abdulaziz University Jeddah Kingdom of Saudi Arabia; ^2^ Department of Otolaryngology–Head and Neck Surgery, Temerty Faculty of Medicine University of Toronto Toronto Ontario Canada; ^3^ Institute of Health Policy, Management and Evaluation, Dalla Lana School of Public Health University of Toronto Toronto Ontario Canada; ^4^ Department of Otolaryngology–Head and Neck Surgery King Faisal Hospital Makkah Kingdom of Saudi Arabia; ^5^ Department of Surgery Faculty of Medicine, Divison of Otolaryngology—Head and Neck Surgery University of British Columbia Vancouver British Columbia Canada; ^6^ Department of Ophthalmology and Otolaryngology, Head and Neck Surgery, Faculty of Medicine Umm Al‐Qura University Makkah Kingdom of Saudi Arabia; ^7^ Department of Surgery King Faisal Specialist Hospital & Research Center Jeddah Kingdom of Saudi Arabia; ^8^ Department of Otolaryngology–Head and Neck Surgery, Division of Rhinology, St. Michael's Hospital Unity Health Toronto Toronto Ontario Canada; ^9^ Department of Otolaryngology–Head and Neck Surgery, Division of Rhinology University of Toronto Toronto Ontario Canada; ^10^ Department of Otolaryngology–Head and Neck Surgery University of Cincinnati College of Medicine Cincinnati Ontario USA

**Keywords:** aged, endoscopic surgical procedure, quality of life, sino‐nasal outcome test, sinusitis

## Abstract

**Objective:**

To assess whether patients of advanced age with chronic rhinosinusitis (CRS) experience comparable symptom improvement and disease control following endoscopic sinus surgery (ESS) compared to younger patients.

**Data Sources:**

Systematic searches were performed across PubMed, Embase, Web of Science, Scopus, and CENTRAL to May 2024.

**Review Methods:**

Following PRISMA guidelines, we included comparative studies with age‐stratified cohorts reporting outcomes for CRS patients undergoing ESS. The primary outcome was symptom improvement measured by the 22‐item Sinonasal Outcome Test (SNOT‐22). Secondary outcomes were disease recurrence and revision rates. Meta‐regression examined the influence of sex, preoperative symptom severity, nasal polyps, and prior ESS.

**Results:**

Across the included studies, data from 3161 patients were analyzed. Advanced‐age patients demonstrated significantly less SNOT‐22 improvement than younger counterparts (SMD −0.36; 95% confidence interval [CI], −0.61 to −0.10; *P* = .01). Nevertheless, recurrence and revision rates were lower in the advanced‐age group—12% and 3%, respectively—corresponding to a 50% reduction in combined risk (RR 0.50; 95% CI, 0.33‐0.75; *P* < .001). Meta‐regression identified male sex as a predictor of greater symptom improvement in advanced‐age patients (*β* = 0.06, *P* = .03), whereas prior ESS was associated with diminished improvement (*β* = −0.05, *P* < .01).

**Conclusion:**

Patients of advanced age may experience less pronounced symptomatic improvement but lower recurrence and revision rates after ESS. These differences should be interpreted with caution, as they may reflect both surgical outcomes and other factors such as comorbidities or reoperation thresholds. Age alone should not preclude ESS, which remains an effective option when tailored to individual patient characteristics.

Chronic rhinosinusitis (CRS) is a prevalent inflammatory condition of the paranasal sinuses that markedly impairs quality of life through persistent nasal obstruction, facial pain, and diminished olfaction, often requiring a surgical intervention for symptomatic relief. Although medical management is typically the first line of treatment, endoscopic sinus surgery (ESS) has become a cornerstone intervention for patients who do not respond adequately to conservative therapies.^1^, ^2^ Despite the overall effectiveness of ESS, it was reported that as many as 20% to 35% of patients who undergo ESS for CRS do not experience significant improvement.^3^, ^4^, ^5^, ^6^

Research indicates that patient age plays a significant role in both the extent of symptom improvement and the likelihood of disease recurrence following surgery.^6^, ^7^, ^8^, ^9^ Notably, the prevalence of CRS rises with age—particularly beyond 50 years—with one epidemiological study of 4098 participants revealing a sharp increase in incidence from the age of 50.^10^, ^11^ This escalating trend with advancing age can be attributed to several factors, including physiological changes such as atrophy of the nasal and paranasal sinus mucosa, reduced mucus production, and impaired mucociliary clearance, all of which may predispose individuals to more persistent and difficult‐to‐manage disease.^12^, ^13^ Accordingly, age 50 represents a clinically and biologically meaningful threshold for investigating age‐related differences in surgical outcomes among patients with CRS. Age‐related variations in tissue healing, immune response, and the presence of comorbidities may further influence postoperative outcomes in terms of symptomatic relief and disease control.^4^, ^5^, ^14^, ^15^ Additionally, preoperative symptom severity and a history of previous ESS have been identified as potential modifiers of surgical outcomes, further complicating the role of age as an independent prognostic factor.^6^, ^16^

In light of the aforementioned observations, there remains a notable gap in the literature posing essential questions about the impact of aging on ESS outcomes. Specifically, patients over 50, with their distinct physiological profiles and associated risk factors, may warrant more focused attention and tailored management strategies. Moreover, there is a notable lack of research addressing the influence of age on disease recurrence following surgery. Gaining a clearer understanding of age‐related effects on ESS outcomes is crucial for refining patient selection, counseling, and perioperative management strategies. Should patients of advanced age experience similar benefits to younger individuals, it would affirm the viability of ESS as an effective treatment for patients of all ages. On the other hand, if aging is found to be linked to diminished efficacy, clinicians may need to adapt their treatment approaches accordingly.

Therefore, the current analysis was designed to rigorously evaluate whether patients beyond 50 years of age differ from their younger counterparts in terms of CRS symptom improvement following ESS. Additionally, it aimed to compare the rates of recurrence and revision surgery between these age groups. We hope the findings of this study will contribute to evidence‐based practice, guiding otolaryngologists in making informed decisions when treating advanced‐age patients with CRS.

## Methods

### Protocol and Registration

This systematic review was conducted in accordance with the Preferred Reporting Items for Systematic Reviews and Meta‐Analyses (PRISMA) guidelines.^17^ Furthermore, the review protocol is registered with the International Prospective Register of Systematic Reviews (ID: CRD420250652152).

### Search Strategy and Study Selection

A thorough search was conducted across 5 electronic databases—PubMed, Embase, Web of Science Core Collection, Scopus, and the Cochrane Central Register of Controlled Trials (CENTRAL)—using a combination of keywords and controlled vocabulary, as outlined in Supplemental Appendix A, available online. This search encompassed all available records from the inception of each database up to May 6, 2024. Studies were considered eligible if they were original articles, regardless of language, and included comparative cohorts that reported outcomes stratified by age, allowing for direct comparison between an older cohort (aged 50 years or beyond) and a younger adult cohort. All included patients were diagnosed with CRS, with or without nasal polyposis, undergoing either primary or revision ESS. Studies were excluded if they involved patients from an undefined older group, had concomitant conditions necessitating ESS, or contained unreliable or unavailable data. Other exclusions were applied to duplicate records, abstract‐only publications, theses, books, preprints, reviews, and conference papers.

The outcomes of interest included improvements in symptoms (as measured by the 22‐item Sinonasal Outcome Test [SNOT‐22]), disease recurrence, and the rate of revision surgery following the index procedure. The definition of disease recurrence was based on the criteria reported in each individual study. Our outcomes of interest were evaluated both within the advanced‐age group and in comparison, to the younger adult population. The final selection of studies was reached by consensus after independent reviewers carefully screened the titles and abstracts of all identified articles.

### Data Extraction

Two reviewers independently extracted data using a standardized form that recorded key details including author names, publication year, study design, sample size, demographics (age and sex), presence of prior ESS, presence of nasal polyps, follow‐up duration/timepoint, preoperative and postoperative SNOT‐22 scores, symptom improvement measured by the SNOT‐22, disease recurrence, and revision surgery after the index procedure. Any discrepancies were resolved through discussion.

### Quality Assessment

The quality of each study was evaluated using the Newcastle‐Ottawa Scale (NOS) for nonrandomized studies.^18^ Two reviewers conducted these assessments independently and in a blinded manner, with any disagreements resolved by discussion. The NOS assesses the quality of non‐randomized studies based on participants selection, group comparability, and outcome assessment.

### Quantitative Synthesis (Meta‐Analysis)

Symptomatic improvement after ESS was quantitatively evaluated using the SNOT‐22. A meta‐analysis was conducted to assess improvements in SNOT‐22 scores (continuous outcome), comparing the changes in scores between advanced‐age (aged 50 years or beyond) and younger cohorts within the first year after surgery—or cases where the mean follow‐up time was within the first year. This outcome's effect size was quantified using the standardized mean difference (SMD). To investigate the factors influencing differences in SNOT‐22 score improvement between advanced‐age and younger cohorts, a meta‐regression was performed, incorporating variables such as male sex, preoperative SNOT‐22 scores, presence of nasal polyps, and prior ESS history. As part of the quantitative synthesis, a sensitivity analysis was planned to explore any potential dose‐response relationship between age and symptom improvement and to address potential heterogeneity arising from the variable age thresholds used across studies.

A subsequent meta‐analysis was carried out regarding disease recurrence and revision surgery (as a single composite dichotomous outcome) within the advanced‐age group in comparison to the younger adult population. The respective effect size was quantified using the risk ratio (RR) estimate. This study outcome was analyzed using logarithmic measure, later back‐transformed to exponential effect size. Heterogeneity was assessed via Cochran's *Q*‐statistic and *I*² statistics, with a random‐effects model applied in cases of statistical heterogeneity. The results were generated using Stata (StataCorp. 2023. Stata Statistical Software: Release 18.; StataCorp LLC). All statistical tests were 2‐sided, and significance was set at *P* < .05.

## Results

### Study Selection

**Figure 1:**
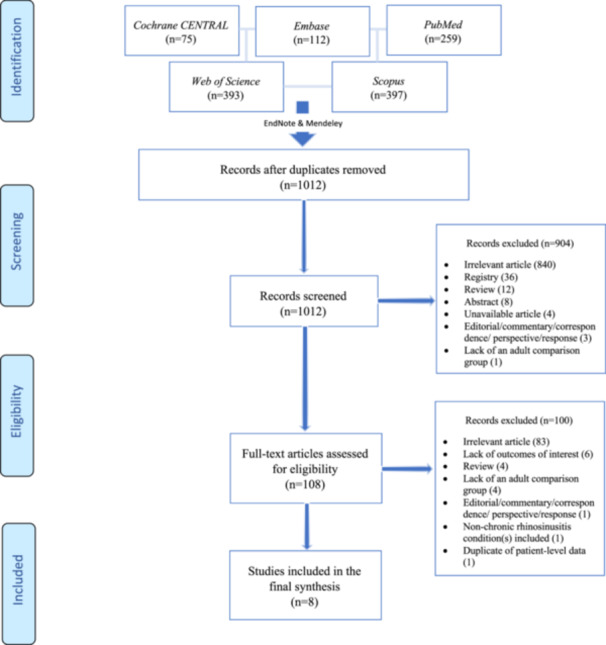
Flow chart depicting the method of study selection.

The search strategy identified 1012 records, from which 8 comparative studies were selected that examined the endpoints relative to the adult population. The process of study selection is depicted in Figure 1.

### Study Characteristics and Quality of the Included Studies

Table 1 summarizes the key features of the included studies, published between 2010 and 2022, of which 7 were retrospective cohort studies. The present review involved 1463 participants in the advanced‐age cohort and 1698 in the comparison group, with the youngest patient being 17 years old.^19^ While all studies provided comparative data between advanced‐age and younger cohorts, the exact age thresholds for defining “advanced‐age” varied: 5 studies used ≥65 years,^7^, ^8^, ^9^, ^19^, ^20^ 2 used ≥60 years,^21^, ^22^ and one defined older patients as ≥50 years.^23^ In studies that reported 3 age strata (eg, 18‐39, 40‐64, ≥65 in Holmes et al^9^; 18‐39, 40‐59, ≥60 in Yancey et al^21^), we included only the youngest and oldest categories in our analysis, excluding the middle‐aged group to minimize overlap and heterogeneity, consistent with our predefined age stratification criteria, which designated 50 years (or older) as the threshold for the advanced‐age cohort.

**Table 1:** Summary Table of the Included Studies

Study ID, year	Design	Cohort (age): mean age (SD or range)	n	Male (%)	Presence of nasal polyps (%)	Prior ESS (%)	Follow‐up time point (months)	Symptom improvement, by SNOT‐22 (Mean ± SD)	Disease improvement, by other scale	Disease control, by recurrence and revision surgery
Brescia et al. 2022^7^	Retrospective cohort	Advanced‐age (≥65): NR	67	65.7	100	NR	48	NR	NR	Recurrence (n = 6)
		Younger (20‐40): NR	105	59.0	100	NR	48	NR	NR	Recurrence (n = 23)
Gardner et al. 2021^19^	Retrospective cohort	Advanced‐age (≥65): 71 (65‐83)	73	52.1	NR	30.6	NR	NR	NR	Revision surgery (n = 2)
		Younger (<65): 45.6 (17–64)	124	39.5	NR	26.6	NR	NR	NR	Revision surgery (n = 5)
Helman et al. 2021^20^	Retrospective cohort	Advanced‐age (≥65): 72.3 (5.2)	67	41.8	55.4 (of n = 65)	36.4 (of n = 66)	≥2–12^a^	% change: −38.1 ± 49.0; n = 35	NOSE (% change: −48.5% ±14.0; n = 6)	NR
		Younger (40‐64): 55.3 (4.3)	24	45.8	36.8 (of n = 19)	19.1 (of n = 21)	≥2–12^a^	% change: −72.9 ± 18.9; n = 13	NOSE (% change: −58.0% ±42.7; n = 13)	NR
Holmes et al. 2020^9^	Retrospective cohort	Advanced‐age (≥65): NR	159	50.9	59.1	0.0	12	preop: 23.5 ± NR; postop: 12.2 ± NR	▪ L–M (preop: 13.7 ± NR; postop: NR) ▪ L–K (preop: 6.0 ± NR; postop: 3.1 ± NR)	NR
		Younger (18‐39): NR	63	49.2	61.9	0.0	12	preop: 33.2 ± NR; postop: 17.4 ± NR	▪ L–M (preop: 13.1 ± NR; postop: NR) ▪ L–K (preop: 5.8 ± NR; postop: 7.6 ± NR)	NR
Crosby et al. 2019^23^	Retrospective cohort	Advanced‐age (≥50): 60.6 (7.8)	634	58.8	52.9	68.1	6	preop: 38.3 ± 22.0; post op: 19.9 ± 16.5; n = 186	NR	NR
		Younger (<50): 36.3 (8.7)	618	51.5	54.8	59.3	6	preop: 43.7 ± 22.8; post op: 22.5 ± 20.9; n = 155	NR	NR
Yancey et al. 2019^21^	Retrospective cohort	Advanced‐age (≥60): NR	131	52.7	33.6	48.1	12 (mean)	▪ preop: 37.6 ± 21.5; postop: 24.2 ± 17.0 ▪ MCID^b^: 66.0%	NR	NR
		Younger (18‐39): NR	100	49.0	34.0	28.0	12 (mean)	▪ preop: 44.1 ± 19.8; post op: 19.2 ± 15.7 ▪ MCID^b^: 83.0%	NR	NR
Lehmann et al. 2018^22^	Prospective cohort	Advanced‐age (60‐80): NR	152	49.3	52.6	55.3	12	preop: 42.7 ± 19.7; change: −20.8 ± 18.5	HUV (preop: 0.8 ± 0.1; change: 0.1 ± 0.12)	NR
		Younger (18‐59): NR	484	45.3	45.7	42.6	12	preop: 49.1 ± 19.8; change: −24.7 ± 19.8	HUV (preop: 0.8 ± 0.2; change: 0.1 ± 0.2)	NR
Ban et al. 2010^8^	Retrospective cohort	Advanced‐age (≥65): 68.6 (65.5‐70)	180	64.4	66.7	10.6	6 ± 7.6 (mean ± SD)	NR	NR	Recurrence (n = 21)
		Younger (16‐64): 40 (27‐50)	180	58.9	77.2	23.3	8.03 ± 14.6 (mean ± SD)	NR	NR	Recurrence (n = 40)

Abbreviations: CI, confidence interval; ESS, endoscopic sinus surgery; HUV, health utility value; L‐K, Lund‐Kennedy; L‐M, Lund‐Mackay; MCID, minimal clinically important difference; NOSE, nasal obstruction symptom evaluation; NR, not reported; SD, standard deviation; SNOT‐22, sinonasal outcome test‐22.

^a^ The time points of ≥2 to 12 months were confirmed in correspondence with the corresponding author.

^b^ It was defined as a reduction of 8.9 or more points in the total SNOT‐22 score.

All participants were diagnosed with CRS, although the proportion of patients with nasal polyposis differed, with one study exclusively focusing on CRS with nasal polyposis (CRSwNP) cases.^7^ Both primary and revision surgeries were evaluated across all studies, and follow‐up periods ranged from 2 to 48 months. Additionally, 7 studies provided data suitable for quantitative synthesis.^7^, ^8^, ^19^, ^20^, ^21^, ^22^, ^23^ With respect to study quality, all studies were graded as NOS of ≥7 to 9. A detailed description of quality assessment is presented in Supplemental Appendix B, available online.

### Symptom Improvement by SNOT‐22

Comparative studies indicated that younger patients often experienced greater SNOT‐22 improvement after ESS in both the short term^20^, ^23^ and long term.^9^, ^21^, ^22^ In short‐term outcomes (≥2 months), Helman et al. found that while both geriatric patients aged ≥65 years and younger controls aged 40 to 64 years improved significantly, the percentage reduction in SNOT‐22 scores was smaller in the geriatric group (−38.1% vs −72.9%, *P* = .0009).^20^ In long‐term follow‐up, Yancey et al reported that although all age groups (18‐39, 40‐59, ≥60 years) improved, elderly patients had the smallest mean change (−15.4) compared with −26.4 in young and −19.2 in middle‐aged adults (*P* = .01).^21^ Holmes et al stratified 431 CRS patients undergoing primary ESS into young adults (18‐39 years), adults (40‐64 years), and elderly (≥65 years). Despite higher preoperative SNOT‐22 scores in young adults (33.2) than adults (25.3) and elderly patients (23.5; *P* = .029), all groups maintained significant improvement for up to 3 years, with no significant age‐related differences at 6 months, 1 year, 2 years, or 3 years.^9^ Similarly, Lehmann et al prospectively evaluated 636 CRS patients aged 18 to 80 years, stratifying outcomes by decade of life. Significant improvements in SNOT‐22 scores were observed across all decades at both 12‐ and 24‐month post‐ESS, with generally comparable gains between age groups. The oldest cohort (70‐80 years) achieved the smallest improvements (−15.7 at 12 months and −15.1 at 24 months).^22^

**Figure 2:**
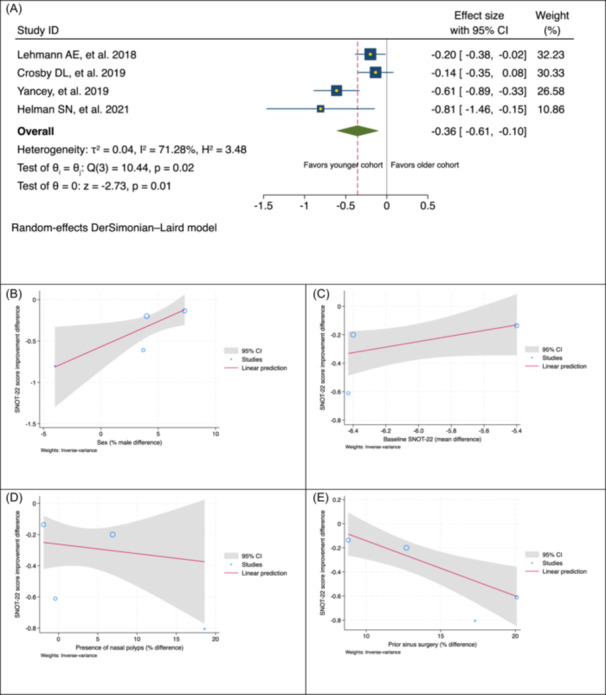
(A) Forest plot showing age‐related difference in SNOT‐22 improvement (advanced age − younger). (B‐E) Meta‐regression by sex, baseline SNOT‐22, presence of nasal polyps, and prior sinus surgery, respectively. CI, confidence interval; SNOT‐22, sinonasal outcome test‐22.

While individual studies varied in their definition of “advanced‐age” patients (≥50, ≥60, or ≥65 years), our primary meta‐analysis pooled all studies comparing patients aged 50 years or beyond with younger cohorts, under a random‐effects model. This revealed a statistically significant difference in SNOT‐22 improvement (SMD −0.36; 95% CI, −0.61 to −0.10; *P* = .01; Figure 2A), less favoring the advanced‐age population. Although 66% of patients in advanced‐age cohorts achieved the minimal clinically important difference (MCID) in SNOT‐22, this remained lower than the 83% observed in younger adults.^21^

Our meta‐regression analysis suggested that higher preoperative SNOT‐22 scores in the younger population, compared to the advanced‐age group, were associated with greater postoperative SNOT‐22 improvement less favoring the advanced‐age cohort. However, this association was not statistically significant (*P* = .14; Figure 2C). Additionally, our regression analysis indicated that the presence of nasal polyps did not significantly influence differences in CRS symptom improvement between the two populations (Figure 2D). Notably, the analysis identified sex and prior ESS history as statistically significant factors influencing CRS symptom improvement. A higher proportion of males was associated with a greater symptom improvement difference favoring the advanced‐age cohort (*β* = 0.06, *P* = .03; Table 2 and Figure 2B). Conversely, a higher proportion of advanced‐age patients with a history of prior ESS was linked to lower SNOT‐22 improvement (*β* = −0.05, *P* < .01; Table 2 and Figure 2E).

**Table 2:** Meta‐Regression Analysis of Factors Contributing to Differences in SNOT‐22 Score Improvements Between Older and Younger Cohorts (Random‐Effects Meta‐Regression)

				95% confidence interval		
Variable	Studies, n	*β* coefficient^a^	SE	Lower	Upper	2‐sided *P‐*value
Sex (male)	4	0.06	0.03	0.01	0.11	.03*
Preoperative SNOT‐22	3	0.19	0.13	−0.07	0.46	.14
Presence of nasal polyps	4	−0.01	0.01	−0.03	0.02	.63
Prior ESS	4	−0.05	0.02	−0.08	−0.02	.003*

Abbreviations: ESS, endoscopic sinus surgery; SE, standard error; SNOT‐22, sinonasal outcome test‐22.

^a^ A positive *β* coefficient indicates that an increase in the covariate is associated with a greater SNOT‐22 improvement difference favoring the advanced‐age cohort, whereas a negative *β* coefficient suggests a greater improvement difference favoring the younger cohort.

* *P* < .05, statistically significant.

### Sensitivity Analysis and Age‐Dependent Effect

As the definition of the “advanced‐age cohort” varied across the included studies, with thresholds ranging from ≥50 to ≥65 years, we conducted a sensitivity analysis to account for this variability and to assess whether higher age cutoffs produced more pronounced outcome differences (Supplemental Appendix C, available online). Using a leave‐one‐out approach, the effect size became more pronounced (SMD −0.47; 95% CI, −0.83 to −0.11; *P* = .011; Supplemental Appendix C, available online) in analyses restricted exclusively to populations aged ≥60 years. These findings suggest a potential dose‐response relationship, wherein increasing age is associated with progressively lower SNOT‐22 improvement following ESS.

### Disease Recurrence and Revision Surgery

Three comparative studies reported on disease recurrence and revision surgery.^7^, ^8^, ^19^ Our proportional meta‐analysis collectively revealed that the disease recurrence rate among the advanced‐age cohort was 12% (95% CI, 8%‐18%) at an average follow‐up of approximately 17 months, with 3% (95% CI, 1%‐11%) undergoing revision surgery after the index procedure. In contrast, the younger population exhibited a recurrence rate of 28% (95% CI, 21%‐38%) at an average follow‐up of approximately 23 months. The pooled analysis demonstrated a statistically significant 50% relative reduction in the risk of disease recurrence and revision surgery in the advanced‐age group compared to younger adults (RR 0.50; 95% CI, 0.33‐0.75; *P* < .001).

## Discussion

### Summary Findings

This systematic review and meta‐analysis evaluated the impact of age on outcomes following ESS in patients with CRS. Our analysis revealed that advanced‐age population, while still benefiting from ESS, demonstrate a statistically significant but modestly reduced improvement in SNOT‐22 scores compared to younger patients. Notably, our analysis identified female sex and prior ESS history as significant factors influencing the difference in SNOT‐22 improvement between the age groups, with their absence being associated with a greater improvement in advanced‐age patients compared to younger population. In parallel, the analysis showed that the rates of disease recurrence and subsequent revision surgery were significantly lower in the advanced‐age cohort, with a pooled risk reduction of 50% compared to younger patients.

### Comparison With Previous Studies

The literature on age‐related differences in ESS outcomes has been heterogeneous. Some earlier studies suggested that older patients might achieve greater symptom improvement—possibly due to higher baseline expectations or differences in disease presentation—while others have reported negligible or even reversed associations when controlling for confounding factors.^6^, ^16^, ^21^ A previous systematic review demonstrated that the older population has more symptom improvement, though it was conducted exclusively among CRSwNP.^6^ A subsequent meta‐analysis study showed that age is insignificantly conversely associated with symptom improvement.^16^ Importantly, these analyses did not account fully for other relevant patient‐ and disease‐related factors, which may contribute to the observed variations in outcomes. Our findings align with the notion that although advanced‐age population exhibit less pronounced symptomatic improvement postoperatively, the durability of surgical benefit—as evidenced by lower recurrence and revision rates—is superior in this age group. Notably, our meta‐regression analysis identified prior ESS history as a significant factor contributing to the differences observed between age groups, suggesting that repeated surgeries may blunt the magnitude of symptomatic improvement.

### Potential Underlying Mechanisms

Several factors may account for the observed discrepancies between symptomatic relief and surgical durability. Age‐related physiological changes—such as mucosal atrophy, impaired mucociliary clearance, diminished tissue regeneration, altered immune responses, and shifts in the nasal microbiome—could lead to a less robust subjective improvement in quality of life despite effective control of objective disease parameters.^5^, ^24^, ^25^, ^26^ Additionally, the higher prevalence of comorbidities and poorer overall health status in the older may limit their functional capacity and subjective perception of improvement following ESS.^14^, ^27^, ^28^, ^29^, ^30^, ^31^ Furthermore, older patients often present with lower baseline SNOT‐22 scores,^9^, ^21^, ^22^, ^23^ which may inherently limit the potential for dramatic score reductions. Soler et al and Fu et al found that low initial SNOT‐22 scores among CRS patients are associated with less improvement.^16^, ^32^ Consistently, advanced‐age patients often have a history of ESS, which may compromise postoperative symptom relief due to modifications in sinus anatomy, scarring, synechiae, and mucosal alterations.^33^ In fact, previous ESS has been associated with less marked improvements in SNOT‐22 scores among the general CRS population.^34^

In addition to these anatomical and symptomatic considerations, emerging evidence suggests that aging is associated with alterations in the inflammatory endotypes of CRS. Specifically, studies have indicated that older patients may experience a shift from type 2 (Th2/eosinophilic) inflammation towards non‐type 2 (neutrophilic or Th1/Th17) profiles. This transition may contribute to a less aggressive disease phenotype, often described as a “burned‐out” form of CRS, characterized by reduced eosinophilic activity and a more stable clinical course. Such endotypic changes could explain the observed lower recurrence and revision rates in advanced‐age patients, despite more modest improvements in subjective symptom scores post‐ESS. These findings align with broader concepts of immunosenescence and inflammaging, where aging leads to a chronic, low‐grade inflammatory state and a decline in adaptive immune responses. In the context of CRS, this may result in diminished type 2 inflammatory responses and a shift towards alternative inflammatory pathways.^5^, ^35^

Conversely, the lower recurrence and revision rates in this population might reflect a combination of factors, including more conservative re‐intervention strategies and a different inflammatory profile that favors stable postoperative outcomes. Our finding suggests that ESS can provide durable long‐term benefits for the population of advanced age, contrasting with some data that reported higher recurrence and revision rates in this age group.^36^ Although we were unable to adjust for potential confounding factors due to data limitations, this result challenges the prevailing notion that older patients are more prone to poorer outcomes after ESS. A significant factor to consider explaining this finding is the differential in quality of and adherence to postoperative care. A study by Tsai et al. observed that older age is associated with better compliance with follow‐up after ESS, indicating potential differences in treatment outcomes based on age.^37^ Further evidence suggests that older patients are more likely to adhere strictly to postoperative care regimens, which impacts long‐term outcomes including recurrence and revision surgery.^38^ A study by Shen et al. demonstrated that adherence to postoperative care is associated with better outcomes, even among patients exhibiting poorer improvement in the early postoperative time. This indicates that the long‐term outcomes following ESS are more influenced by the quality of postoperative care than the short‐term outcomes.^39^ These previous studies perfectly align with our findings; while older patients may experience a smaller short‐term improvement, their long‐term outcomes, including disease recurrence and revision surgery rates, are comparable to or even better than those of younger adults. These insights add to the existing body of knowledge by highlighting the potential for ESS to yield favorable long‐term outcomes in carefully selected and well‐managed older individuals.

### Clinical Implications

These results have important implications for clinical practice. ESS remains an effective treatment modality for CRS across age groups. For advanced‐age patients, the expectation of modest symptomatic improvement should be balanced against the potential for lower recurrence and revision rates. Clinicians should incorporate these findings into preoperative counseling, emphasizing that while the subjective improvement might be less pronounced, the long‐term stability of surgical outcomes appears favorable. However, these apparent long‐term advantages should be interpreted cautiously, as they may be partly influenced by nondisease‐related factors such as surgical decision‐making thresholds and comorbidities affecting operative candidacy.

### Limitations and Future Directions

Several limitations warrant consideration. The majority of the studies included in this meta‐analysis were retrospective, which may introduce selection bias and limit causal inference. Variability in surgical techniques and patient populations further complicates direct comparisons across studies. Additionally, limited data on potential confounding variables—such as detailed comorbidity profiles, medication use, and specific sinonasal conditions—restricted our ability to fully adjust for these factors. Another limitation is the variability in age cut‐offs used to define the advanced‐age cohort across studies (≥50, ≥60, and ≥65 years), which may contribute to heterogeneity in outcome interpretation. Although we attempted to address this through sensitivity analysis, the lack of standardized age stratification across the literature remains a constraint. Future prospective studies with consistent age groupings—ideally decade‐based—combined with standardized outcome measures and longer follow‐up, are needed to validate and expand upon these findings. Moreover, further research into the biological mechanisms underlying age‐related differences in ESS outcomes could provide insights for tailoring perioperative management strategies.

### Conclusion

In summary, despite the rising incidence of CRS beyond the age of 50, our meta‐analysis suggests that patients of advanced‐age may experience less pronounced symptomatic improvement following ESS but may have lower rates of disease recurrence and revision surgery. ESS can provide meaningful benefit in this age group, and with appropriate selection, individualized management, and realistic counseling, ESS remains a viable treatment option for advanced‐age CRS patients.

## Author Contributions

**Ahmad A. Mirza**, acquisition of the data, formal analysis, interpretation of the data, and drafting the work; **Hussam A. Senan**, acquisition of the data and drafting the work; **Juan Carlos Hernaiz‐Leonardo**, interpretation of the data and critically reviewing the work; **Osama A. Marglani**, conception of the work and critically reviewing the work; **John M. Lee**, conception of the work and critically reviewing the work; **Ahmad R. Sedaghat**, conception of the work and critically reviewing the work; **Amin R. Javer**, conception of the work, design of the work, and critically reviewing the work; all authors approved the final version for publication, and all agree to be accountable for all aspects of the work.

## Disclosures

### Competing interests

None.

### Funding source

None.

## Supporting information

Supporting information.

Supporting information.

Supporting information.
