# Supplementary material for: Advanced Age in Sinus Surgery: Diminished Symptom Gains but Enhanced Surgical Durability in Chronic Rhinosinusitis
Source: Otolaryngol Head Neck Surg. 2025 Oct 14;173(6):1348–58. doi: 10.1002/ohn.70043 (PMC12661474; doi:10.1002/ohn.70043)
Supplement: Supplementary file 1 — Supporting information. [file OHN-173-1348-s003.docx]

**Appendix A.** Results of systematic search in different databases

*Cochrane Central Register of Controlled Trials (CENTRAL)*

| **#** | **Search** | **Results** |
| --- | --- | --- |
| 1 | (sinusitis) OR (rhinosinusitis):ti,ab,kw | 15,001 |
| 2 | (elderly) OR (geriatric):ti,ab,kw OR (older):ti,ab,kw | 131,532 |
| 3 | ("Endoscopic Sinus Surgery") OR (ESS):ti,ab,kw OR (Operati*):ti,ab,kw | 125,697 |
| 4 | #1 AND #2 AND #3 | 75 Trials |

*Embase Classic + Embase (Ovid)*

| **#** | **Search** | **Results** |
| --- | --- | --- |
| 1 | exp chronic sinusitis/ or exp sinusitis/ | 58,786 |
| 2 | elderly.mp. or exp aged/ | 4,110,804 |
| 3 | exp geriatric patient/ or exp geriatrics/ | 82,935 |
| 4 | endoscopic sinus surgery/ | 6,844 |
| 5 | "quality of life".mp. or exp "quality of life"/ | 846,866 |
| 6 | #2 OR #3 | 4,139,593 |
| 7 | #1 AND #4 AND #5 AND #6 | 112 |

*PubMed*

| **#** | **Search** | **Results** |
| --- | --- | --- |
| 1 | "paranasal sinuses"[MeSH Terms] OR ("paranasal"[All Fields] AND "sinuses"[All Fields]) OR "paranasal sinuses"[All Fields] OR "sinuses"[All Fields] OR "sinusal"[All Fields] OR "sinuse"[All Fields] OR "sinusitis"[MeSH Terms] OR "sinusitis"[All Fields] OR "sinusitides"[All Fields] OR ("rhinosinusal"[All Fields] OR "rhinosinusitis"[MeSH Terms] OR "rhinosinusitis"[All Fields]) | 75,914 |
| 2 | "Endoscopic Sinus Surgery"[All Fields] OR "ESS"[All Fields] | 16,137 |
| 3 | "aged"[MeSH Terms] OR "aged"[All Fields] OR "elderly"[All Fields] OR "elderlies"[All Fields] OR "elderly s"[All Fields] OR "elderlys"[All Fields] OR "geriatric"[All Fields] OR "geriatrics"[MeSH Terms] OR "geriatrics"[All Fields] OR "older"[All Fields] OR "olders"[All Fields] | 6,394,394 |
| 4 | "quality of life"[All Fields] | 466,918 |
| 5 | "effective*"[All Fields] OR "outcome*"[All Fields] OR "efficacies"[All Fields] OR "efficacious"[All Fields] OR "efficaciously"[All Fields] OR "efficaciousness"[All Fields] OR "efficacy"[All Fields] | 6,128,652 |
| 6 | #1 AND #2 AND #3 AND #4 AND #5 | 259 |

*Scopus*

| **#** | **Search** | **Results** |
| --- | --- | --- |
| 1 | *TITLE-ABS-KEY ( sinusitis OR rhinosinusitis )* | 59,875 |
| 2 | *TITLE-ABS-KEY ( elderly OR geriatric OR older )* | 1,673,678 |
| 3 | *TITLE-ABS-KEY ( "Endoscopic Sinus Surgery" OR "ESS" OR "Operati*" )* | 5,182,378 |
| 4 | #1 AND #2 AND #3 | 397 |

*Web of Science core collection*

| **#** | **Search** | **Results** |
| --- | --- | --- |
| 1 | (ALL=(sinusitis)) OR ALL=(Rhinosinusitis) | 29,878 |
| 2 | ((ALL=(elderly)) OR ALL=(Geriatric)) OR ALL=(older) | 2,513,950 |
| 3 | (ALL=("Endoscopic Sinus Surgery")) OR ALL=(ESS) | 39,026 |
| 4 | #1 AND #2 AND #3 | 393 |
