# Supplementary material for: Advanced Age in Sinus Surgery: Diminished Symptom Gains but Enhanced Surgical Durability in Chronic Rhinosinusitis
Source: Otolaryngol Head Neck Surg. 2025 Oct 14;173(6):1348–58. doi: 10.1002/ohn.70043 (PMC12661474; doi:10.1002/ohn.70043)
Supplement: Supplementary file 2 — Supporting information. [file OHN-173-1348-s002.docx]

**Appendix B.** Quality assessment of the included studies

| **NEWCASTLE - OTTAWA QUALITY ASSESSMENT SCALE FOR COHORT STUDIES** | | | | | | | | | |
| --- | --- | --- | --- | --- | --- | --- | --- | --- | --- |
| **Study ID, year** | **SELECTION** | | | | **COMPARABILITY** | **OUTCOME** | | | **Total score** |
|  | 1. Representativeness of the exposed cohort | 2. Selection of the non-exposed cohort | 3.  Ascertainment of exposure | 4.  Demonstration that outcome of interest was not present at start of study | Comparability of cohorts on the basis of the design or analysis | 1.  Assessment of outcome | 2.  Was follow-up long enough for outcomes to occur? | 3.  Adequacy of follow up of cohorts |  |
| Brescia G, et al. 2022 | * | * | * | * | ** | * | * | * | 9 |
| Gardner JR, et al. 2021 | * | * | * | * | ** | * |  |  | 7 |
| Helman SN, et al. 2021 | * | * | * | * | ** | * |  |  | 7 |
| Holmes T, et al. 2020 | * | * | * | * | ** | * | * | * | 9 |
| Crosby DL, et al. 2019 | * | * | * | * | ** | * | * |  | 8 |
| Yancey KL, et al. 2019 | * | * | * | * | ** | * | * |  | 8 |
| Lehmann AE, et al. 2018 | * | * | * | * | * | * | * | * | 8 |
| Ban JH, et al. 2010 | * | * | * | * | ** | * |  |  | 7 |
