# Supplementary material for: Advanced Age in Sinus Surgery: Diminished Symptom Gains but Enhanced Surgical Durability in Chronic Rhinosinusitis
Source: Otolaryngol Head Neck Surg. 2025 Oct 14;173(6):1348–58. doi: 10.1002/ohn.70043 (PMC12661474; doi:10.1002/ohn.70043)
Supplement: Supplementary file 3 — Supporting information. [file OHN-173-1348-s001.docx]

**Appendix C.** Leave-one-out sensitivity analysis for age-related differences in SNOT-22 improvement

|  |
| --- |

**p*-value at <0.05, statistically significant.

**CI:** confidence interval
